# Leaching of Copper Concentrates with Iodized Salts in a Saline Acid Medium: Part 2—Effect on Chloride Concentration and an Aerated System

**DOI:** 10.3390/ma16175940

**Published:** 2023-08-30

**Authors:** César I. Castellón, María E. Taboada

**Affiliations:** 1 Departamento de Ingeniería en Minas, Facultad de Ingeniería, Universidad de Antofagasta, Av. Angamos 601, Antofagasta 1240000, Chile; 2 Departamento de Ingeniería Química y Procesos de Minerales, Facultad de Ingeniería, Universidad de Antofagasta, Av. Angamos 601, Antofagasta 1240000, Chile

**Keywords:** chalcopyrite, leaching, iodide, chloride, mechanism, air

## Abstract

To enhance the leaching of chalcopyrite concentrates, this study evaluated a new process for extracting copper using iodized solutions and sulfuric acid diluted in seawater without pressure or high temperatures. The work involved a leaching test carried out under various conditions by varying the concentrations of chloride ions, H_2_SO_4_, and an evenly distributed oxygen supply in an aeration system. It was demonstrated that Cl^−^ ion addition could promote the chalcopyrite-leaching process. The leaching efficiency of copper reached 70% after 96 h. However, a chloride ion dosage excess can have the opposite effect on extraction, reducing copper recovery. XRD and SEM-EDS results showed that cuprous chloride (CuCl) was formed at high dosages (>0.5 M); meanwhile, at a lower dosage, elemental sulfur (S) was formed in the presence of sulfuric acid solution and seawater medium. In contrast, in an aerated system, surface roughness markedly increased due to continuous oxidation on the surface of the ore. This change in morphology and the high value of the redox potential, given by the aerated system and the acidic environment, allowed copper recovery of up to 70% after 96 h. The results showed that an aerated system is the most effective factor in chalcopyrite concentrate leaching.

## 1. Introduction

Chalcopyrite mineral (CuFeS_2_) is one of the most significant sulfides, and approximately 70% of the world’s copper resources are hosted in this mineral [1]. Furthermore, around 80% of the known copper occurs in copper porphyry deposits and sediments [2], commonly processed with smelting operations of copper concentrates [3]. The extractive metallurgy of chalcopyrite predominantly relies on a conventional pathway encompassing comminution and flotation, smelting, and electro-refining, accounting for approximately 85% of copper production in Chile [4]. The hydrometallurgical extraction of copper from this chalcopyrite mineral is considered to be a more environmentally friendly operation compared with the use of pyrometallurgical extraction, especially for copper sulfide minerals of low grades [5]. However, processing via the hydrometallurgical route is limited, both in oxidative and reductive processes, due to characteristics such as the refractory nature of the mineral during leaching [6] and its main limitation related to mineral passivation caused by the formation of elemental sulfur, polysulfide species, hydroxy sulfate species of iron, or a combination of these [7,8,9,10].

Various hydrometallurgical processes of the primary copper mineral have been carried out to reduce leaching times and increase its dissolution rate. Currently, the hydrometallurgical leaching process of chalcopyrite can be divided into three categories: oxidative leaching, coordination leaching, and bioleaching. For some of the main parameters studied in this regard, researchers have conducted tests with the addition of different concentrations of chloride to leaching systems and even by using direct seawater, desalinated water, or hypersaline water [11,12,13,14,15,16,17,18,19,20,21,22,23]. Additionally, the effect of oxygen addition to the system has been studied as a parameter to break the passivation layer and reduce the leaching times of the mineral [24,25,26,27].

### 1.1. Use of Chloride Ions in the Leaching of Chalcopyrite

The development of chloride-mediated coordination leaching, although pioneered decades ago, has gained greater relevance in recent times due to its attractive features and its capacity to treat copper concentrates in a less environmentally detrimental manner.

Some of the main advantages offered by the use of chloride ions in leaching systems are (i) higher leaching kinetics under atmospheric conditions; (ii) catalytic effect of chloride ions due to the formation of metal complexes; (iii) high solubility of copper and iron in chloride solutions depending on pH [15,28]; and (iv) the fact that pH is maintained at a desired value for a longer time. In addition to the above, the effects of chloride ions on the mineral surface not only result in higher copper extraction rates but also increase the porosity on the mineral surface, reducing the tendency for chalcopyrite passivation [16,18,29,30]. The increased porosity generated by chloride solutions reduces the diffusion barrier between the sulfur formed on the mineral surface and the leaching agents. Lu, Jeffrey [16] demonstrated this in chalcopyrite concentrates, where leaching heaps with chloride solutions formed a porous crystalline sulfur layer, unlike heaps without chloride solutions, where an amorphous or cryptocrystalline sulfur layer is formed. This crystalline and porous layer allows for better access of oxidants to the mineral surface.

The presence of chlorides in a sulfuric acid medium, in addition to providing improved kinetics and oxidation of sulfur to its elemental form, tends to form complex chloro-copper species (known as chloro-complexes) that modify the redox potentials, allowing for lower oxidation potentials required for chalcopyrite leaching (540 mV) compared with the typically required potential of over 750 mV in a sulfate medium [13]. The formation of chloro-complexes, which positively affects the leaching chemistry, exhibits higher anodic activity and induces changes in surface morphology [18]. The ability of chloride ions to form complexes is crucial. Berger and Winand [31] have proposed the following classification of the strength of Cl^−^ receptors: AgCl > CuCl > PbCl_2_ > ZnCl_2_ > CuCl_2_ > FeCl_3_ > FeCl_2_ > NiCl_2_ > HCl, NaCl, KCl (Cl^−^ donors). While cuprous chloride (CuCl) is sparingly soluble in water, its solubility increases in the presence of chlorides [32]. In contrast, as the chloride concentration increases, the solubility of Cu^2+^ decreases [33].

Table 1 displays the thermodynamic values of complex formation when cuprous and cupric ions are present in the solution (Reactions (1)–(7)) [34,35,36,37].

According to the generation of cupric chloro-complexes from the previous table, only CuCl^+^ and CuCl_3_^−^ are thermodynamically feasible and could dissolve chalcopyrite, as indicated by Reactions (9) and (10).

(8) $${CuFeS}_{2}+{3Cu}^{2+}+{4Cl}^{-}\to 4CuCl+{Fe}^{2+}+2S\;∆{G^{{}^{\circ}}}_{25\;{}^{\circ}C}=-51.20\mathrm{kJ}$$

(9) $${CuFeS}_{2}+{3CuCl}_{3}^{-}\to 4CuCl+{Fe}^{2+}+{5Cl}^{-}+2S\;∆{G^{{}^{\circ}}}_{25\;{}^{\circ}C}=-91.94\mathrm{kJ}$$

(10) $${CuFeS}_{2}+{3CuCl}^{+}+{Cl}^{-}\to 4CuCl+{Fe}^{2+}+2S\;∆{G^{{}^{\circ}}}_{25\;{}^{\circ}C}=-44.61\mathrm{kJ}$$

According to this thermodynamic analysis, it is likely that precipitated CuCl is formed as a product of the reactions. Furthermore, the formed CuCl, in the presence of oxygen and an acidic medium, dissolves according to reaction (11), generating the leaching reagent CuCl^+^. This reaction has a value of ΔG° = −267.86 kJ.

(11) $$4CuCl+{4H}^{+}+O_{2}\to {4CuCl}^{+}+{2H}_{2}O\;∆{G^{{}^{\circ}}}_{25\;{}^{\circ}C}=-267.86\mathrm{kJ}$$

### 1.2. Use of an Aerated System

However, corrosion under moderate chloride conditions has been one of the disadvantages of its use in this type of coordination leaching, which is why the oxidative leaching of minerals using air or oxygen is currently a widely utilized technique in the extractive industry for extracting valuable metals. Oxygen plays an essential role in this process as it aids in dissolving metals in the leaching solution.

Nevertheless, under normal pressure conditions, the oxygen present in the atmosphere does not possess the necessary potential to enable the leaching of copper present in chalcopyrite. Therefore, it is necessary to use other oxidizing agents to achieve the appropriate redox potential for the leaching process.

In the case of chalcopyrite mineral, the presence of oxygen significantly enhances the copper dissolution rate by regenerating ferric ions (Fe^3+^) [38,39]. 

The objective of this study is to evaluate the effects of chloride concentrations and an aerated system on copper recovery involving the addition of potassium iodide (KI) as an oxidizing agent in an acidic medium with seawater. The use of this type of iodide salt dissolves sulfide minerals due to the high proton activity generated by the mixture of acid and oxygen. In addition, the chloride ions provided by seawater aid in the kinetics of mineral leaching. Additionally, this study aims to further investigate the characterization and morphological changes that occur due to the addition of these variables in the leaching of copper concentrates, allowing the surface of chalcopyrite mineral to be attacked, resulting in the creation of cracks or a porous surface that enables the leaching solution to penetrate and exchange ions.

## 2. Materials and Methods

### 2.1. Mineral Sample and Reagents

**Figure 1:**
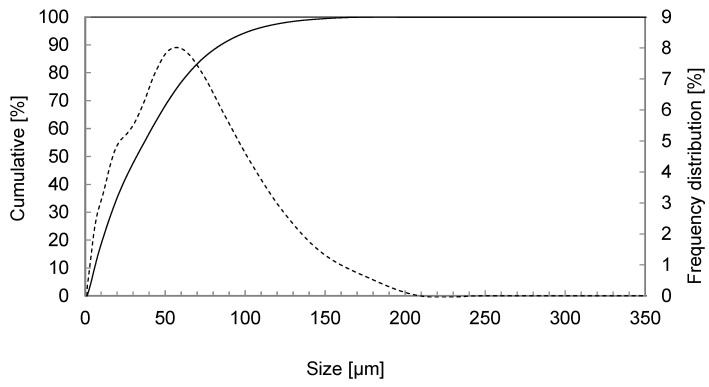
Particle-size distribution of the concentrates (solid line: cumulative %; dotted line: Frequency distribution %). The y-axis represents the cumulative percentage; the secondary y-axis represents the frequency distribution percentage of particle size; and the x-axis represents the particle size in micrometers.

Chalcopyrite concentrates for the tests were obtained from a Chilean mining company. Figure 1 displays the particle-size distribution of chalcopyrite concentrates, which is 100% below a size of 296.0 µm (100%—#50) and P_80_ = 60.66 µm. The particle-size distribution was determined using laser diffraction Microtrac S3500 (Microtrac, York, PA, USA).

**Figure 2:**
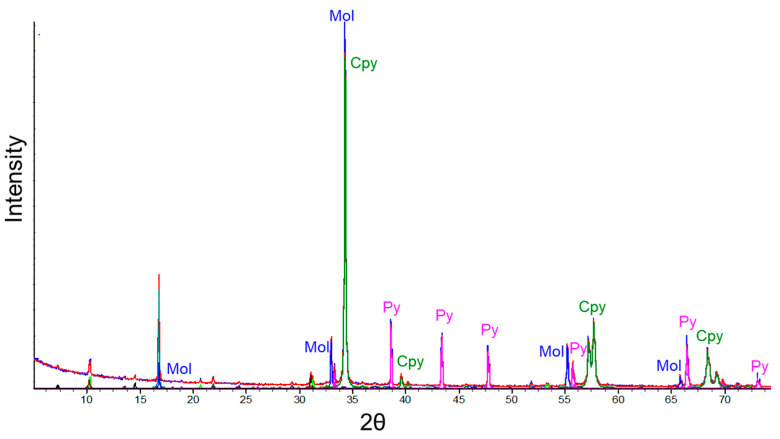
XRD of chalcopyrite concentrates (Cpy = chalcopyrite; Py = piryte; Mol = molibdenyte).

Furthermore, the chemical composition of the concentrates was determined using inductively coupled plasma atomic emission spectroscopy (ICP-AES), while the mineralogical content was determined with the quantitative evaluation of materials using a scanning electron microscope (QEMSCAN), model Zeiss EVO 50 (Zeiss, Oberkochen, Germany). The results are shown in Table 2, where according to the mineralogical information and the XRD (X-ray diffraction) spectrum obtained with Shimadzu XRD 6100 equipment (Shimadzu Corporation, Kyoto, Japan) (Figure 2), the concentrates are primarily composed of chalcopyrite (61.51 wt.%) and pyrite (23.3 wt.%), with quantities of covellite, chalcocite, sphalerite, and molybdenite. Meanwhile, the gangue mineral is predominantly composed of quartz, dolomite, muscovite, and albite. The chemical analysis in Table 2 shows that the concentrates primarily consist of Cu, Fe, and S. Based on these data, the sample is composed of 63% copper sulfides, followed by 24.3% other sulfides. Similarly, the soluble copper in the sample was determined to be 1.24%.

**Figure 3:**
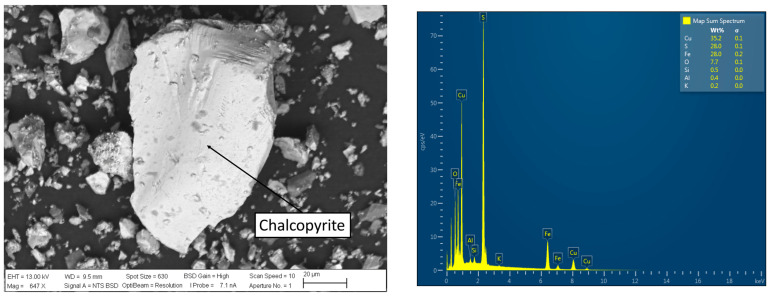

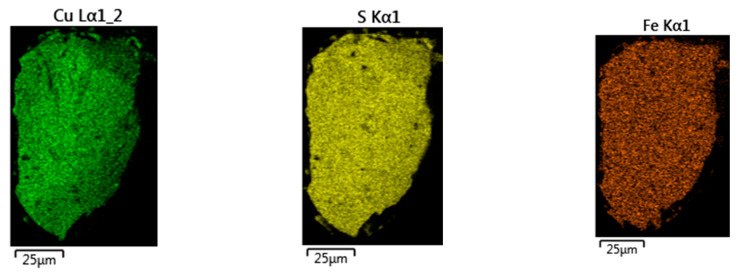
SEM and EDS micrograph of chalcopyrite concentrate.

Furthermore, Figure 3 presents a microphotograph obtained with scanning electron microscopy (SEM) analysis using Zeiss EVO MA10 model equipment (CARL ZEISS Ltd., Oberkochen, Germany) and an energy-dispersive X-ray analyzer (EDS) on a point of the concentrates at 647× magnification (OXFORD Instruments, Oxford, UK). According to this analysis, it can be observed that the chalcopyrite particles do not exhibit a high specific surface area or a high BET content (Brunauer, Emmett, and Teller), as rough particles are not prominently visible.

Potassium iodide (99.0% absolute; Merck, Darmstadt, Germany), sulfuric acid (95–97%; Merck, Darmstadt, Germany), and sodium chloride (99.9%; Merck, Darmstadt, Germany) of analytical grade were used in leaching tests. Seawater was obtained 200 m from the coast in San Jorge Bay, Antofagasta, Chile. The seawater (pH = 7.1) was passed through a quartz sand filter (50 µm) and a mechanical polyethylene filter (1 µm) to remove insoluble particulate matter. Its composition is shown in Table 3.

### 2.2. Procedure of the Leaching Experiments

Following the same methodology as in the first part [40], concentrate-leaching tests were performed in 2 L jacketed glass reactors. Each reactor was loaded with 1 L of leaching solution (sulfuric acid, seawater, iodide potassium, and sodium chloride) and was sealed with film to avoid evaporation. Once the solution reached the desired temperature, 50 g of solid sample was added to the reactor. Before the leaching tests, the concentrate sample was washed with distilled water and acetone (C_3_H_6_O) with the purpose to remove any remaining flotation reagents used in the process of concentration. The pulp was stirred to obtain a homogenous mix using a propeller at a rotation speed of 450 rpm. A 10 mL aliquot of solution was taken periodically during the test for copper analysis using the Atomic Absorption Spectroscopy method (AAS). Redox potential (ORP) and pH were measured throughout the test with a portable Hanna meter model HI991003 (HANNA, Woonsocket, RI, USA). All experiments were performed in triplicate. The solid residues were carefully filtered, washed with distilled water, and dried at 60 °C to constant weight. Samples for mineralogical characterization and particle-size determination were taken.

**Figure 4:**
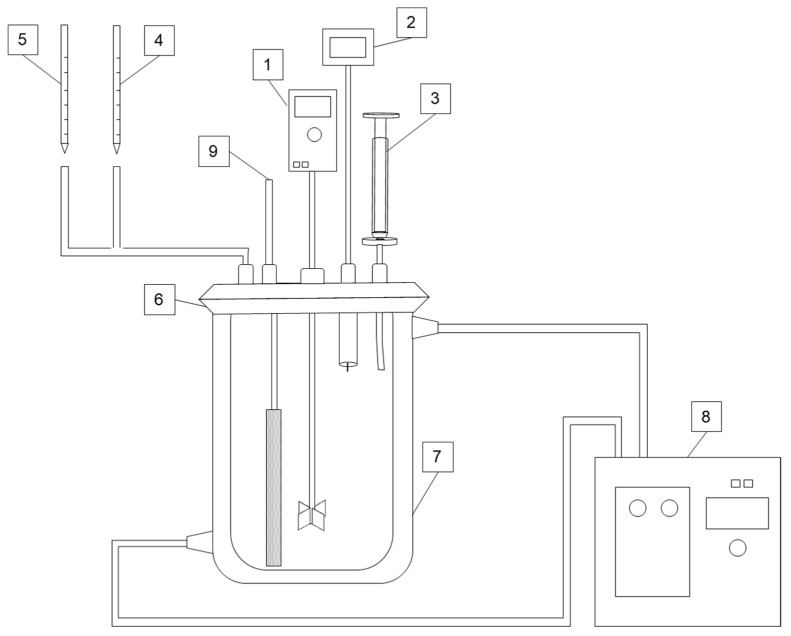
The schematic diagram of the leaching setup: 1. overhead stirrer; 2. pH and ORP probe; 3. sampling tube, equipped with syringe and syringe filter; 4. H_2_SO_4_; 5. oxidant; 6. sealed lid; 7. jacketed reactor; 8. water bath; 9. air pump with sponge filter.

To study the effect of chloride ions on chalcopyrite leaching, the following was performed: In addition to the conditions mentioned above, the leaching experiments were conducted for a leaching time of 96 h. The leaching solution used was direct seawater with an approximate chloride concentration of 20 g/L. Additional amounts of 5, 10, 25, 50, and 100 g/L were added to seawater to obtain final concentrations of 25, 30, 45, 70, and 120 g/L, respectively. In the case of an aerated system, the airflow was obtained using a pump that supplied an airflow of 3 L/min. Before meeting the solution, the air was dispersed into small bubbles, typically > 1 mm, using an air diffuser with a height of 15 cm to maximize contact with the solution. A schematic representation of this process is shown in Figure 4.

## 3. Results and Discussion

### 3.1. Influence of Water Type

To initially determine the effect of using direct seawater (~19 g/L Cl^−^) on the use of fresh water (~0 g/L Cl^−^), tests were conducted by varying the following conditions: KI = 100 ppm, H_2_SO_4_ = 0.1, and 0.5 M, with T = 45 °C. 

**Figure 5:**
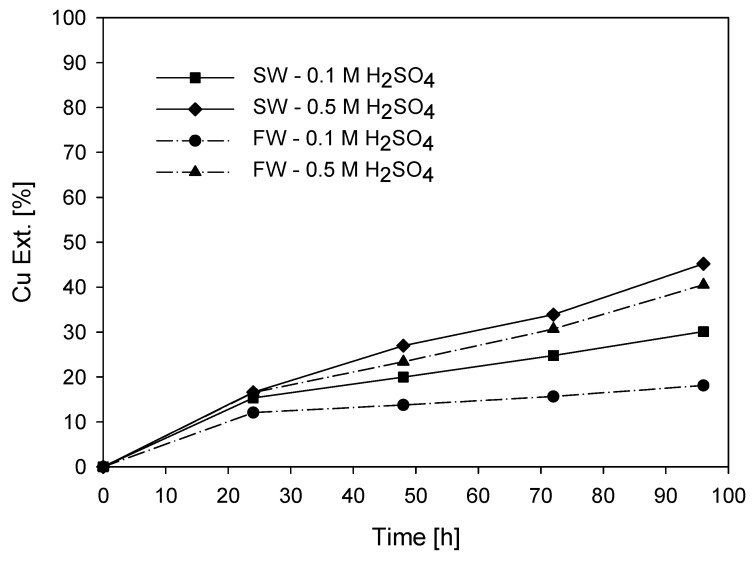
Cu Ext. (%) vs. time (h) with seawater (SW) and freshwater (FW). (■) = SW and 0.1 M H_2_SO_4_; (◆) = SW and 0.5 M H_2_SO_4_; (●) = FW and 0.1 M H_2_SO_4_; (▲) = FW and 0.5 M H_2_SO_4_. Conditions: KI= 100 ppm, T= 45 °C, 50 g of sample with 1000 mL of solution, stirring rate = 450 rpm.

According to Figure 5, the extraction curves after 40 h show favorable extraction when the system incorporates chloride ions from seawater. Leaching is more effective with chloride due to its higher kinetic rate of electron transfer [21,22,41,42]. In addition to the effect of chloride ion addition, acid plays an important role in leaching systems. As shown in the previous figure, higher acidity concentration results in higher extraction. In these experiments, the effect of increasing acid concentration by 40 times is greater (0.1 and 0.5 M) than the effect of increasing chloride concentration by 19 times (between 0 and 19 g/L). At low acid concentrations (0.1 M), copper extraction is more favorable with the integration of chloride ions from seawater, resulting in a 12% increase compared with tests at higher acid concentrations (0.5 M), where the increase is only 3%.

Regarding the potential, the solutions start with values close to 680 mV (vs. Ag/AgCl) and then stabilize within ranges around 600 mV (vs. Ag/AgCl) as leaching progresses. Towards the end of the test, there is a slight increase in these values. At the end of the test, the potential values range from 590 to 630 mV (vs. Ag/AgCl) indicating an oxidizing environment where copper transfer to the solution can occur. As for the pH values, at an acid concentration of 0.5 M, they indicate a highly acidic environment, remaining close to 0. In contrast, systems with acid molarity of 0.1 M maintain pH values between 0.8 and 1.4. In the system with acid molarity of 0.1 M and seawater, as the leaching time progresses, subtle increases are observed, indicating that the acid is consumed during the process. The pH values with seawater and 0.1 M acid show a slight increase over time due to the reactions involving the present chloride (Cl^−^). This allows the acid to be consumed, while chlorine is oxidized and released as gaseous Cl_2_.

### 3.2. Influence of Chloride Concentration (Cl^−^) without Oxidant

**Figure 6:**
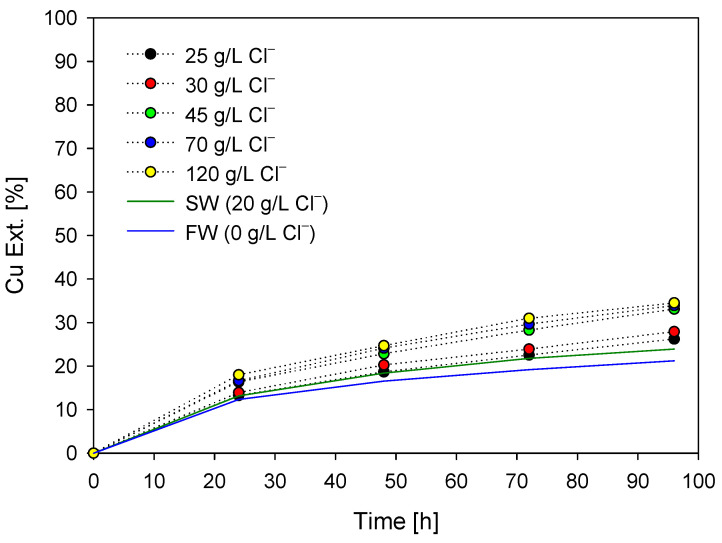
Cu Ext. (%) vs. time (h) with chloride ion addition into seawater (SW) and freshwater (FW). Conditions: H_2_SO_4_ = 0.5 M, T = 45 °C, 50 g of sample with 1000 mL of solution (without oxidant), stirring rate of 450 rpm.

To assess the extent to which chloride ions enhance the leaching of concentrates, the sample was initially leached without the addition of an oxidant. Figure 6 shows the effect of increasing chloride concentrations on copper leaching from chalcopyrite concentrates. An acidity concentration of 0.5 M was used for all tests, as it has shown the best extraction results in previous studies [20,21,22,40]. The extraction results obtained using only freshwater (FW) and seawater (SW) (without chloride addition) are also included. 

According to the results, an increase in chloride concentration leads to an increase in copper dissolution from the concentrates, with an optimal value being observed at 45 g/L Cl^−^ (which corresponds to the 20 g/L Cl^−^ from seawater and an additional 25 g/L Cl^−^). Furthermore, it can be observed that copper dissolution with 70 g/L and 120 g/L Cl^−^ does not show a substantial change at the end of the test.

Similarly, the curve with seawater and fresh water in the system becomes asymptotic, while the curves with the presence of chloride ions follow a constant dissolution rate. This indicates that Cu^+^ and Cu^2+^ ions are thermodynamically stable species, and along with iron ions, they can form complexes with chloride ions and act as oxidizing agents, according to reactions (1) and (5). Therefore, as the initial chloride ion concentration increases, the dissolution of copper from a chalcopyrite concentrate also increases [15]. On the other hand, it is observed that an increase in chloride above 45 g/L results in higher copper extraction. However, these types of extraction lack economic viability, as only marginal increases of 1.03% and 1.05% in copper recovery are achieved when using 74 g/L and 120 g/L chloride, respectively. Therefore, this slight improvement in copper recovery translates into a significant rise in salt addition. Thus, it can be concluded that the optimal addition of chloride in the system is 45 g/L, resulting in 30% extraction of copper instead of 20% when using seawater alone.

### 3.3. Influence of Chloride Concentration (Cl^−^) with Oxidant

**Figure 7:**
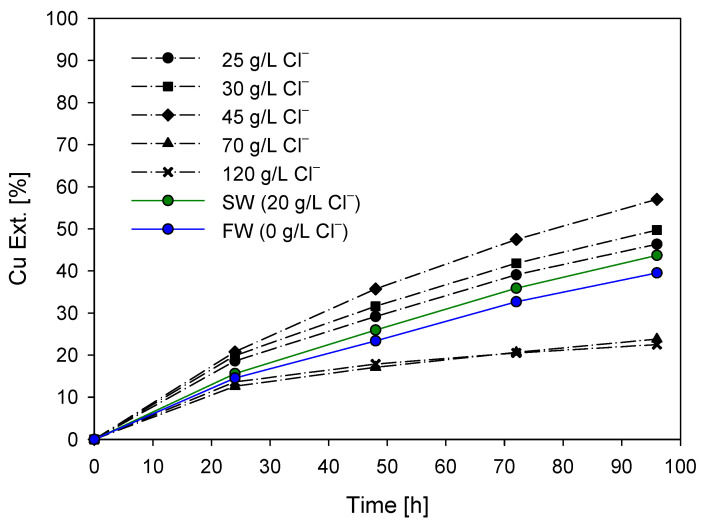
Cu Ext. (%) vs. time (h) with chloride ion addition (blue dot: FW; green dot: SW). Conditions: KI = 100 ppm, H_2_SO_4_ = 0.5 M, T = 45 °C, 50 g of sample with 1000 mL of solution, stirring rate of 450 rpm.

The leaching rate of copper from the concentrates was studied using solutions containing 0.5 M of H_2_SO_4_, an oxidant concentration of 100 ppm of KI, and increasing chloride concentrations at 45 °C. The chloride concentrations used were 5, 10, 25, 50, and 100 g/L Cl^−^ and were added to seawater (~20 g/L Cl^−^). Figure 7 shows the results of the extraction curves obtained with the addition of chloride.

According to the graph, generally, a higher chloride concentration allows for greater copper extraction because in saline and alkaline environments, chalcopyrite and bornite are more susceptible to oxidation, which is consistent with the literature [15,43]. However, this increase is observed only up to chloride doses below 70 g/L. According to Figure 7, the presence of free chloride in the initial leaching medium, considering the use of seawater, increases copper extraction, with copper extraction of 57.0% being achieved with the addition of 45 g of chloride ions, and 43.7% extraction, when no additional chloride is used in the leaching system. This improvement in leaching is attributed to the increased presence of chloride ions, which facilitate the breakdown of the chalcopyrite sulfide matrix [44]. Furthermore, the use of only freshwater without any presence of chloride ions allows for copper extraction of only 39.5%, which is mainly attributed to the oxidative strength of the acid and iodide present. Thus, in the absence of chloride ions, the dissolution of chalcopyrite is lower.

**Figure 8:**
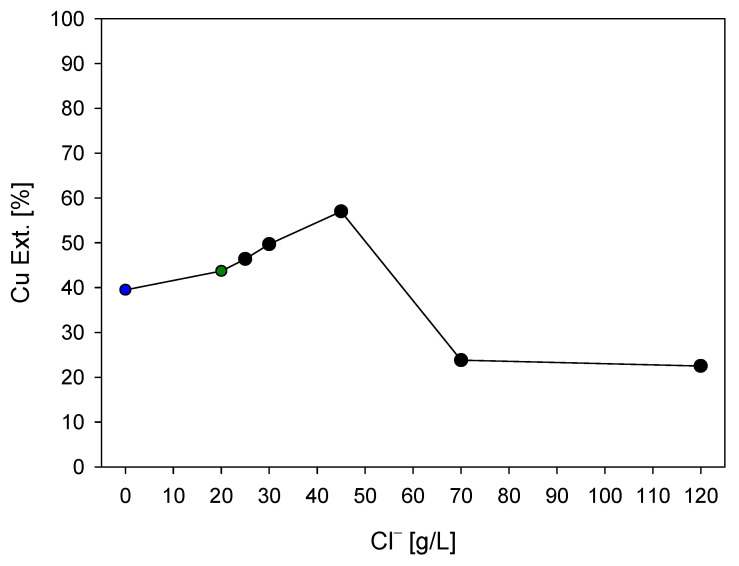
Cu Ext. (%) vs. Cl^−^ (g/L) at 96 h (blue dot: FW; green dot: SW). Conditions: KI = 100 ppm, H_2_SO_4_ = 0.5 M, T = 45 °C, 50 g of sample with 1000 mL of seawater solution, stirring rate of 450 rpm.

Figure 8 illustrates the final copper extraction at 96 h of leaching with different total concentrations of chloride ions in the solution. It can be observed that an increase in the chloride ion concentration leads to an improvement in extraction. However, this increase has a limit (between 45 and 70 g/L chloride ions), beyond which the extraction dramatically decreases. This decrease in the percentage of copper extraction with an increase in chloride addition has been reported by Lu, Jeffrey [16], Ruiz, Montes [45], who established that chloride concentrations higher than 0.5 M do not influence the leaching of chalcopyrite. Furthermore, Palmer, Nebo [46] indicated that the leaching rate increases with concentration if the addition of sodium chloride is low but becomes independent of concentration for values higher than one molar. Figure 6 and Figure 7 show that the dissolution rate does not appear to be affected by the total concentration of chloride ions when concentrations higher than 50 g/L chloride ions are used. These results confirm the preliminary observations that the presence of chloride ions is necessary to enhance the leaching kinetics, but high chloride concentrations are not essential under the studied conditions.

One possible reason for this decrease in copper extraction at higher concentrations beyond the optimal dose is that the free chloride present in the solution reacts with the copper ions in the aqueous phase. The release and presence of both ions allow for the formation of a copper complex; in this case, according to speciation models, the complex formed is the CuCl^+^ ion (reaction (1)), which precipitates as cuprous chloride (CuCl) (reaction (12)).

(12) $$CuCl^{+}+e^{-}\to CuCl\;∆{G^{{}^{\circ}}}_{45\;{}^{\circ}C}=-52.546\mathrm{kJ}$$

**Figure 9:**
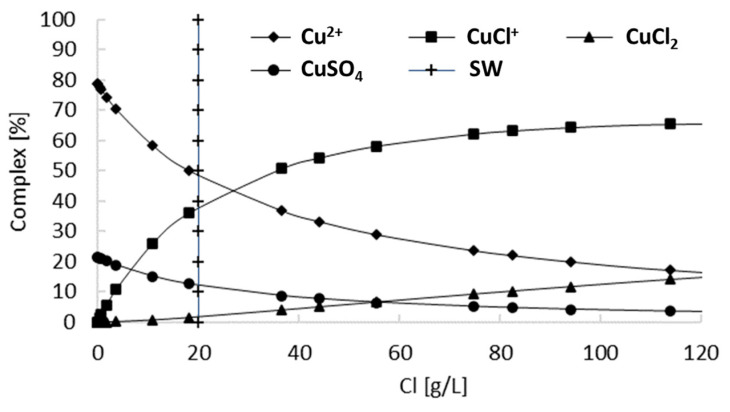
Speciation curve of Cu^2+^ chloride complexes at 25 °C. Conditions: pH = 5 and 0.131 mM of Cu^2+^. Modified from Chesne and Kim [47].

Figure 9 presents a copper speciation model within the experimental range [33,47], which predicts that for a given copper concentration, an increase in chloride concentration significantly decreases the concentration of Cu^2+^ while increasing the concentration of the chloride complex and results in the thermodynamically feasible reactions (8) and (10). According to the model, the predominant species in a solution with seawater (20 g/L Cl^−^) is Cu^2+^ (uncomplexed) at nearly 50%, followed by CuCl^+^ at 35%. Thermodynamically, based on the formation energy from the species table (see Table 1), an increase in chloride concentration in the solution (with the addition of chloride ions) increasingly favors the formation of this complex (CuCl^+^) [47].

Therefore, and according to Figure 7 and Figure 9, a concentration above a specific level of chloride ions in the leaching of chalcopyrite mineral is attributed to factors related to the thermodynamics and kinetics of the process, where at certain chloride concentrations, a significant portion of copper may be present in the solution as chloro-complexes, in this case, CuCl. When equilibrium is reached between the mineral and the complex, further increasing the chloride concentration may not contribute to improved copper leaching kinetics; hence, the rate becomes independent of additional chloride ion additions under conditions of high chloride concentration.

### 3.4. Analysis of the Chalcopyrite Surface after Chloride Addition

**Figure 10:**
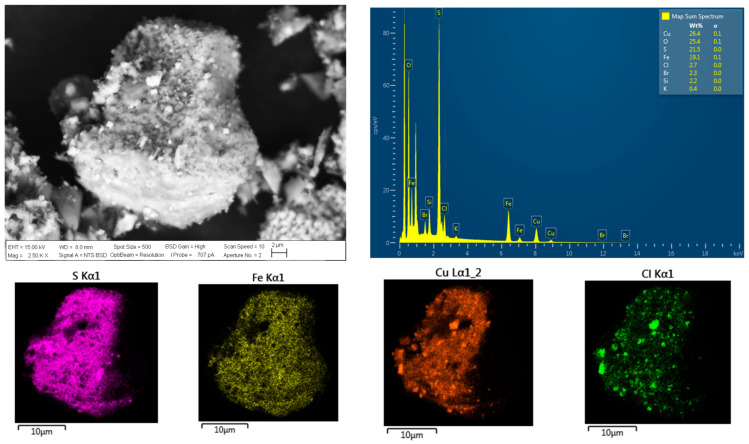
SEM and EDS microanalyses of a leached chalcopyrite particle with addition of Cl^−^ at a concentration of 70 g/L. Conditions: 100 ppm of KI, 45 °C, 50 g of sample with 1000 mL of seawater solution, stirring rate of 450 rpm.

**Figure 11:**
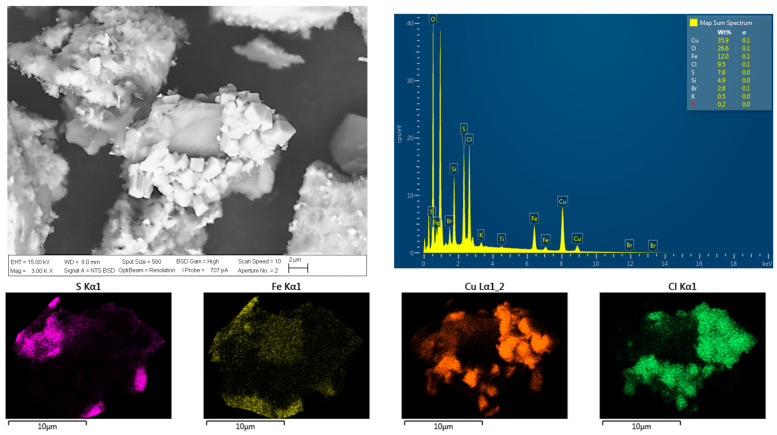
SEM and EDS microanalyses of a leached chalcopyrite particle with addition of Cl^−^ at a concentration of 120 g/L. Conditions: 100 ppm of KI, 45 °C, 50 g of sample with 1000 mL of seawater solution, stirring rate of 450 rpm.

The particles of chalcopyrite were investigated using SEM and EDS analyses (see Figure 10 and Figure 11) to visualize the porosity on the surface area to understand the effect of chloride ion presence on the surface properties of the residue, as well as the precipitation of chloride salts on the surface of chalcopyrite grains. These salts correspond to cuprous chloride (CuCl) according to the thermodynamic analysis of reactions (1), (5), and (11), which coincides with a typical cubic crystal structure of cuprous chloride. According to these images, the formation of precipitates is more pronounced, as observed in the SEM analysis, when higher concentrations of chloride are present in the solution. It can be observed that the surface area and porosity of the residue are greater in the presence of a higher concentration of chloride ions, in contrast to the original concentrates shown in Figure 3. Greater porosity facilitates the diffusion of reactants and products to and from the reaction sites. SEM analysis reveals that chloride ions promote the formation of porous layers of elemental sulfur on the mineral surfaces, thereby enhancing the extraction of Cu.

**Figure 12:**
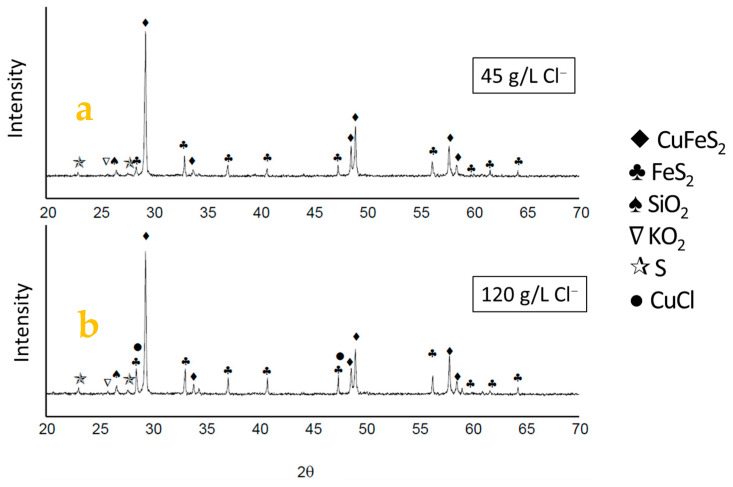
Characteristic diffractograms of the samples with (**a**) 45 g/L Cl^−^ and (**b**) 120 g/L Cl^−^. Conditions: 100 ppm of KI, 45 °C, 50 g of sample with 1000 mL of seawater solution, stirring rate of 450 rpm.

The leached residues from the samples using 45 g/L and 120 g/L chloride ions were analyzed using XRD, as shown in Figure 12. XRD was performed on these two samples as they exhibited the highest copper extraction and were the ones where chloride had a limited effect on leaching. An increase in the intensity of pyrite peaks is observed with the addition of 25 to 100 g/L chloride to the solution, along with the presence of the crystalline phase of CuCl in the sample with the highest chloride concentration, which is confirmed by the SEM-EDS analyses and the above speciation curve, indicating the precipitation of this salt on the grain surfaces. The presence of another oxidizing agent could prevent the precipitation of CuCl. Similarly, XRD revealed the presence of elemental sulfur in both residues. 

Since EDS analysis and XRD did not confirm the presence of jarosite as a reaction product, it is suggested that the main reaction products were CuCl and elemental S°.

### 3.5. Effect of an Aerated System on the Leaching of Copper Concentrates

**Figure 13:**
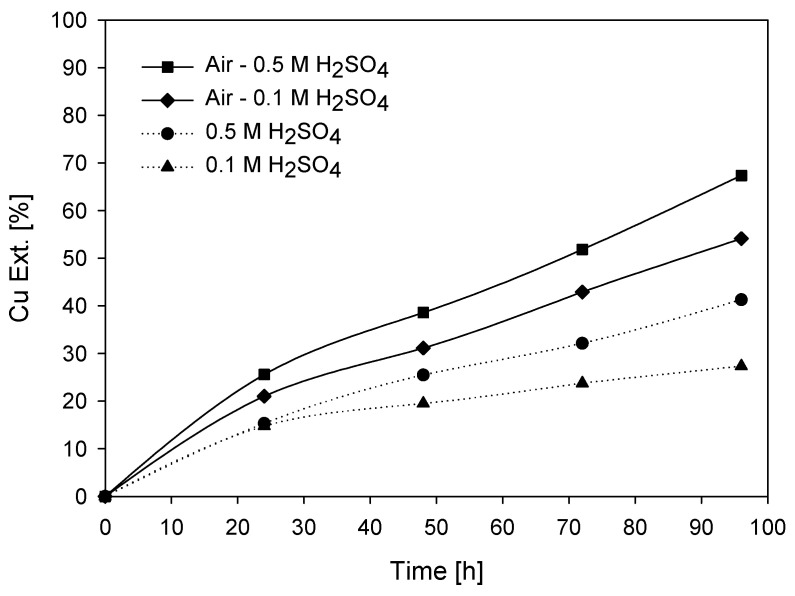
Cu Ext. (%) vs. time (h) with aerated system. (■) = air and 0.5 M H_2_SO_4_; (◆) = air and 0.1 M H_2_SO_4_; (●) = 0.5 M H_2_SO_4_; (▲) = 0.1 M H_2_SO_4_. Conditions: 100 ppm of KI, 45 °C, 50 g of sample with 1000 mL of seawater solution, stirring rate of 450 rpm.

Another variable included in the leaching tests, in addition to the concentrations of H_2_SO_4_ and KI, is the addition of a specific volume of air. The leaching process for this type of test involved a combination of mechanical and pneumatic agitation (Pachuca-type), where the latter involves injecting air from the bottom of the reactor, resulting in high turbulence in the pulp. The injected air acts as an oxidizing agent, where oxygen promotes the dissolution of target species. In the tests, acid concentrations of 0.1 and 0.5 M with KI = 100 ppm were used as the oxidant concentrations at 45 °C. Air addition was carried out using a pump that supplied an airflow of 3 L/min to enhance the leaching kinetics. Figure 13 shows the results obtained for the aerated systems.

It is clearly observed that the systems with air are favored, in both systems with 0.1 M and 0.5 M sulfuric acid. That is due to the interaction of oxygen with the solution and mineral, which enhances the leaching kinetics. In this case, the leaching of chalcopyrite can proceed in two steps. In the first or initial stage (reaction (13)), the principal chemical reaction is mineral oxidation via the oxygen reduction reaction. With the progression of leaching, the soluble concentrations of Cu and Fe increase due to their extraction and are released into the solution. The ferrous species (Fe^2+^) formed via reaction (13) can be oxidized by oxygen to generate ferric species (Fe^3+^) (reaction (14)), which can further oxidize the mineral (reaction (15)). As ferric species are now involved in the leaching of chalcopyrite, copper extraction increases in the final stage [48]. However, it is widely recognized that slow leaching kinetics can be encountered in this final stage, as many studies have shown that the rate-limiting step for copper extraction is the transport process through the formed sulfur layer [29,41,49].


(13) $${\mathrm{CuFeS}}_{2}+O_{2}+4H^{+}\to 2{\mathrm{Cu}}^{2+}+{\mathrm{Fe}}^{2+}+2\;S^{\circ }+2H_{2}O$$

(14) $$4{\mathrm{Fe}}^{2+}+O_{2}+4H^{+}\to 4{\mathrm{Fe}}^{3+}+2H_{2}O$$

(15) $${\mathrm{CuFeS}}_{2}+4{\mathrm{Fe}}^{3+}\to {\mathrm{Cu}}^{2+}+5{\mathrm{Fe}}^{2+}+2\;S^{\circ }$$

**Figure 14:**
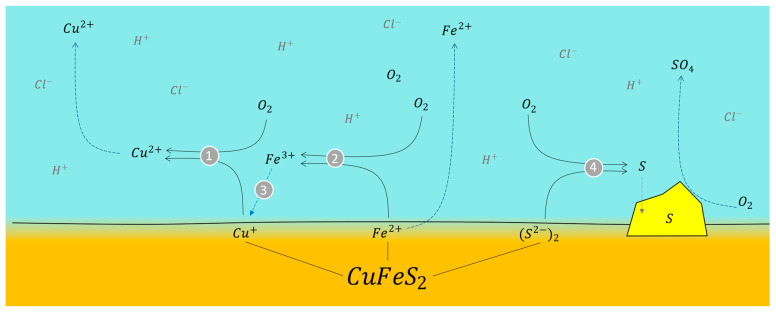
Schematization of the reaction between oxygen and chalcopyrite ore in an acidic environment. Conditions: 100 ppm of KI, 45 °C, 50 g of sample with 1000 mL of seawater solution, stirring rate of 450 rpm.

Figure 14 depicts the reaction mechanism between chalcopyrite mineral and oxygen in an acidic environment. At point 1, the Cu^+^ ion, released into the solution by the oxidant, undergoes oxidation to Cu^2+^ by losing an electron to the oxygen present. Similarly, at point 2, the ferrous ion (Fe^2+^) is oxidized to a ferric ion (Fe^3+^), acting as an additional oxidant in the system. This oxidation process facilitates the release of the copper ion into the solution, while oxygen (O_2_) is reduced to a superoxide ion (O_2_^−^). Simultaneously, the sulfide ion reacts with oxygen, resulting in the production of sulfur, which precipitates onto the mineral’s surface. Under conditions of high oxidation (with a substantial oxygen supply), the reaction between elemental sulfur and oxygen generates sulfuric acid [50]. The formation of sulfates from sulfuric acid arises from a series of reactions. Following the generation of elemental sulfur on the surface of chalcopyrite mineral and facilitated by the presence of dissolved oxygen in the solution, sulfur dioxide (SO_2_) is obtained with the reaction of sulfur with oxygen. Subsequently, sulfur dioxide is further oxidized to transform into sulfur trioxide (SO_3_), which, when dissolved in water, eventually gives rise to sulfuric acid (H_2_SO_4_).

Therefore, according to the above, the presence of oxygen during chalcopyrite leaching allows for the regeneration of active oxidants. According to the results of the redox potential for both experiments, it remains at values close to 600 mV (vs. Ag/AgCl) for both systems. The potential of the system with 0.5 M sulfuric acid shows a slightly higher value than the system with 0.1 M sulfuric acid, which is due to the higher acidity creating a more aggressive environment for chalcopyrite mineral. Similarly, oxygen allows for favorable copper extraction, but its excessive use from the bottom of the reactor and a particle size below 200 mesh cause sulfides (including chalcopyrite) to adhere to the bubble due to the hydrophobic nature of the mineral and its ambient oxidation [51]. The hydrophobicity of sulfides has been attributed to oxidation reactions on the particle surface, which result in higher exposure of sulfur from Cu-S and Fe-S bonds. This sulfur is highly hydrophobic, and even the release of copper and iron ions contributes to the hydrophobicity necessary for adherence to bubbles.

The pH values obtained from both systems (with air and without air) showed that in the case of induced aeration with a concentration of 0.1 M acid, the pH steadily increased, reaching a value close to 1.7 after 96 h. On the other hand, with a concentration of 0.5 M acid, the pH remained constant and close to zero throughout the test. The systems without induced air showed similar pH behavior with both acid concentrations.

Based on the results shown, it can be observed that air is a much more influential factor than sulfuric acid in copper extraction [40].

### 3.6. Surface Analysis of Chalcopyrite in an Aerated System

**Figure 15:**
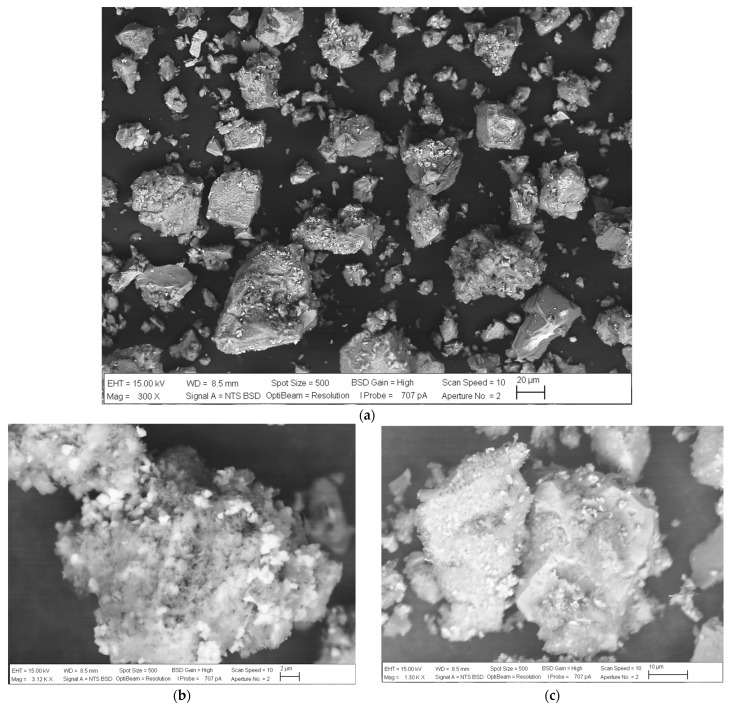

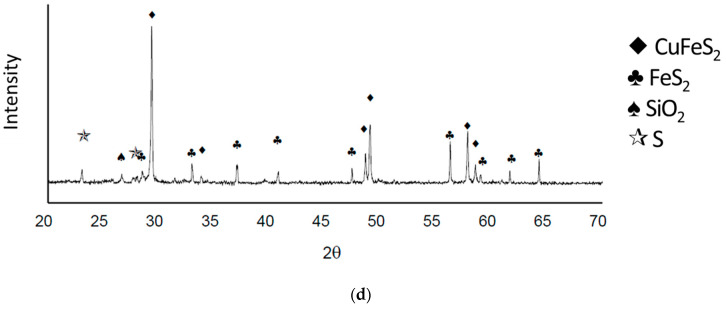
SEM and XRD analyses of tailing with the use of air and 0.5 M of H_2_SO_4_. (**a**) General SEM microanalysis; (**b**,**c**) general microanalysis of chalcopyrite grains; and (**d**) XRD. Conditions: 100 ppm of KI, 45 °C, 50 g of sample with 1000 mL of seawater solution, stirring rate of 450 rpm.

To assess the surface characteristics of the leached mineral, an SEM analysis was performed on the tailings with the use of air and 0.5 M H_2_SO_4_. The objective was to observe any potential alterations in the morphology or texture of the concentrate grains. The findings of this analysis are overall depicted in Figure 15. Initially, Figure 15a presents an image of the tailings at a magnification of 300×, offering an perspective. Notably, the particles exhibit sponge-like precipitates on their surfaces. Figure 15b,c provide a more detailed examination of the chalcopyrite grain morphology and their response to the introduction of air. Based on the XRD analysis presented in Figure 15d, it can be observed that the layer formed on the chalcopyrite particles is composed of elemental sulfur. No other mineral phases were detected in the analysis. Since there were no significant oxidative conditions present to generate SO_4_^2−^, the addition of air to the solution resulted in the formation of a crystalline sulfur layer. This aligns with the mechanism proposed by Mojtahedi, Rasouli [48], which is consistent with the results proposed in the present study. Therefore, in an aerated system with oxygen, reactions (13) and (15) were the oxidation reactions for the initial and subsequent stages of chalcopyrite mineral, consistently with previous studies [29,52,53].

In addition to oxygen, the presence of Cl^−^ ions from seawater allows for increased metal solubility in the leaching system and the formation of a porous layer due to the sulfur-containing products. This porous layer facilitates the diffusion of reactants and improves the rates of copper extraction and recovery [18,19,41,54].

## 4. Conclusions

- The presence of chloride ions is crucial to enhancing leaching kinetics. However, it is concluded that concentrations greater than 45 g/L chloride are not necessary to achieve this effect.
- A chloride ion concentration of 45 g/L in the leaching solution allows for an improved leaching rate of chalcopyrite concentrates in the presence of potassium iodide, resulting in a maximum copper recovery of 57%.
- According to SEM-EDS and XRD analyses, the presence of a high chloride dosage resulted in significant precipitation of the CuCl compound on chalcopyrite grains, along with a lesser amount of elemental sulfur and moderate porosity. This is because the chloride ion, at high doses, is freely available in solution and reacts with Cu^2+^ ions. Additionally, with the addition of 25 g/L chloride, no CuCl compound was detected, and only a few precipitates of elemental sulfur were detected.
- Treating chalcopyrite ore with the addition of air as an additional oxidant achieves high copper extraction of approximately 70% in a shorter leaching time. According to surface analyses using SEM, the presence of air in the interstices of the grains contributes to the formation of sponge-like elemental sulfur on the surface, creating high porosity, which facilitates ion exchange within the solution.

## Figures and Tables

**Table 1:** Formation of copper chloro-complexes (HSC, v 6.0).

Reaction	ΔG° 25 °C (kJ)	
Cupric		
Cu^2+^ + Cl^−^ = CuCl^+^	−2.197	(1)
CuCl^+^ + Cl^−^ = CuCl_2_	6.650	(2)
CuCl_2_ + Cl^−^ = CuCl_3_^−^	−2.431	(3)
CuCl_3_^−^ + Cl^−^ = CuCl_4_^2−^	13.133	(4)
Cuprous		
Cu^+^ + Cl^−^ = CuCl	−13.302	(5)
CuCl + Cl^−^ = CuCl_2_^−^	−16.945	(6)
CuCl_2_^−^ + Cl^−^ = CuCl_3_^2−^	−2.292	(7)

**Table 2:** Chemical and mineralogical analysis of chalcopyrite concentrates.

Chemical Analysis		Mineralogical Analysis		
Element	wt.%	Mineral	Formula	wt.%
Si	2.3	Quartz	SiO_2_	2.2
Fe	32.7	Pyrite	FeS_2_	23.3
Al	1.0	Chalcopyrite	CuFeS_2_	61.5
Mg	0.2	Covellite	CuS	1.5
Ca	0.3	Molybdenite	MoS_2_	0.4
Cu	25.0	Dolomite	CaMg(CO_3_)_2_	1.4
Zn	0.5	Boehmite	AlOOH	2.0
Ti	0.5	Chalcanthite	CuSO_4_•5H_2_O	1.2
Mo	0.2	Albite	NaAlSi_3_O_8_	2.2
K	0.4	Muscovite	KAl_2_(Si_3_Al)O_10_(OH)_2_	1.9
C	0.2	Biotite	K(Mg, Fe^2+^)_3_(Si_3_Al)O_10_(OH)_2_	0.7
Na	0.3	Sphalerite	(Zn_x_, Fe_1−x_)S	0.6
S	36.3	Gypsum	CaSO_4_•2H_2_O	0.8
		Clinochlore	(Mg, Fe^2+^)_5_Al(Si_3_Al)O_10_(OH)_8_	0.2

**Table 3:** Major composition of seawater from San Jorge Bay, Chile.

Ion	Concentration (mg/L)
${Na}^{+}$	9950
${Mg}^{2+}$	1250
${Ca}^{2+}$	400
$K^{+}$	380
${Cl}^{-}$	19,450
${HCO}_{3}^{-}$	150

## Data Availability

The data presented in this study are available on request from authors C.I.C and M.E.T.
